# ProphET, prophage estimation tool: A stand-alone prophage sequence prediction tool with self-updating reference database

**DOI:** 10.1371/journal.pone.0223364

**Published:** 2019-10-02

**Authors:** João L. Reis-Cunha, Daniella C. Bartholomeu, Abigail L. Manson, Ashlee M. Earl, Gustavo C. Cerqueira

**Affiliations:** 1 Infectious Disease and Microbiome Program, Broad Institute of MIT and Harvard, Cambridge, Massachusetts, United States of America; 2 Departamento de Parasitologia, Instituto de Ciências Biológicas, Universidade Federal de Minas Gerais, Belo Horizonte, Minas Gerais, Brazil; 3 Personal Genome Diagnostics, Baltimore, Maryland, United States of America; Northwestern University Feinberg School of Medicine, UNITED STATES

## Abstract

**Background:**

Prophages play a significant role in prokaryotic evolution, often altering the function of the cell that they infect via transfer of new genes e.g., virulence or antibiotic resistance factors, inactivation of existing genes or by modifying gene expression. Recently, phage therapy has gathered renewed interest as a promising alternative to control bacterial infections. Cataloging the repertoire of prophages in large collections of species’ genomes is an important initial step in understanding their evolution and potential therapeutic utility. However, current widely-used tools for identifying prophages within bacterial genome sequences are mainly web-based, can have long response times, and do not scale to keep pace with the many thousands of genomes currently being sequenced routinely.

**Methodology:**

In this work, we present ProphET, an easy to install prophage predictor to be used in Linux operation system, without the constraints associated with a web-based tool. ProphET predictions rely on similarity searches against a database of prophage genes, taking as input a bacterial genome sequence in FASTA format and its corresponding gene annotation in GFF. ProphET identifies prophages in three steps: similarity search, calculation of the density of prophage genes, and edge refinement. ProphET performance was evaluated and compared with other phage predictors based on a set of 54 bacterial genomes containing 267 manually annotated prophages.

**Findings and conclusions:**

ProphET identifies prophages in bacterial genomes with high precision and offers a fast, highly scalable alternative to widely-used web-based applications for prophage detection.

## Introduction

Prophages play an important role in the evolution of bacterial genomes and their pathogenicity [1–3]. Sequence alterations resulting from a prophage integration event can change or knock out gene functions, as well as alter gene expression [1–3]. In addition, they can contribute to pathogenicity by transferring virulence and antibiotic resistance factors into a genome [4–8]. Because of the development of resistance to conventional antibiotic therapeutics, the use of phage therapy as a tool to combat bacterial infections has gathered renewed interest from the scientific community and public health decision makers as a promising alternative to antibiotics [9].

Cataloging the repertoire of prophages and their integration sites is an important initial step in understanding their clinical implications, either as a contributor to bacterial virulence, or as a therapeutic tool. While a handful of prophage identification tools have been designed [4,10,11], successful prediction of prophages from bacterial genomes is hampered by the extraordinary diversity, low similarity and extensive genome size variation (which can range from 3 to 497 kb) across phage families [8,12]. Among the few publicly available prophage prediction tools, Prophinder [13], PHAST [14], PHASTER [15] and PhySpy [16] are the most well-known. Prophinder, PHAST and PHASTER are only available as web applications, which limits their throughput, and routinely burdens users with long response times during periods of high demand. This limits applicability for analysis of large datasets consisting of thousands of bacterial genomes, which are increasingly commonplace. Thus, we sought to design a stand-alone prophage prediction tool that does not have the constraints associated with a web-based tool. Herein, we present ProphET (Prophage Estimation Tool) a fast, easy to use, scalable stand-alone application that identifies prophages in bacterial genomes. In benchmarking, ProphET performed comparably or with higher precision than Prophinder, PHAST, PHASTER and PhySpy, and allowed prophage predictions in a shorter or comparable time compared to currently used tools.

## Material and methods

### ProphET repository

ProphET is a Linux stand-alone application that can be freely downloaded at https://github.com/jaumlrc/ProphET. Instructions regarding installation, input files formats, tutorials on how to run ProphET and a test dataset can be found at this link.

### Phage sequence database

ProphET predictions rely on similarity searches against a database of phage genes, which is generated automatically during ProphET’s installation. The ProphET database is built from the predicted proteome sequences of phage genomes available in GenBank, belonging to 18 families listed in Krupovic 2011 [17]. We excluded protein sequences annotated as ABC transporters, as these are also highly conserved in non-prophage containing regions of both prokaryotes and eukaryotes genomes, and thus could lead to false-positive phage predictions [18]. The current version contains 142,575 protein sequences representing the proteome of 1,435 phages (from bacterial and archaeal, DNA and RNA bacteriophages, with linear, circular or segmented genomes). However, as novel bacteriophage genomic sequences are deposited in GenBank, the database size will increase. The database can be manually updated by executing the update command from ProphET’s home directory. A file reporting the database download date is copied to the results directory of every ProphET execution, and all previous database instances, including the download date and the number of coding sequences per phage family are stored, allowing for reproducibility of results.

### Input files

ProphET takes two input files: a bacterial genome sequence in FASTA format and its corresponding gene annotation in General Feature Format (GFF). The GFF file must be formatted as specified by the Sequence Ontology Consortium (https://github.com/The-Sequence-Ontology/Specifications/blob/master/gff3.md). However, ProphET also provides a GFF converter, as part of GFFLib (package in ProphET setup), allowing the user to convert other GFFs to a format that can be used by ProphET. Instructions on how to run the converter are provided in ProphET GitHub (https://github.com/jaumlrc/ProphET).

### Phage prediction

**Fig 1:**
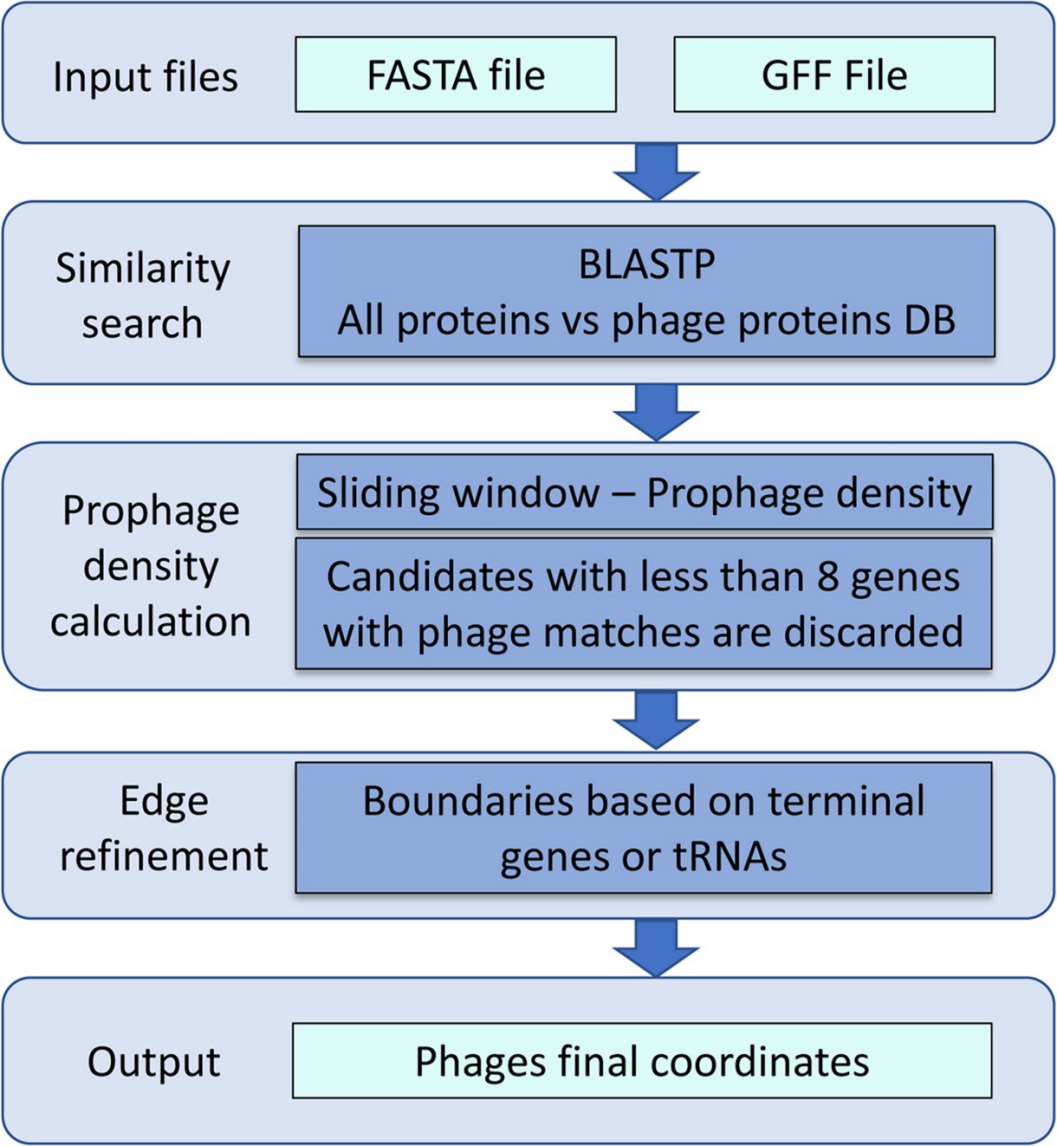
ProphET pipeline.

ProphET identifies prophages based on the density of phage-like genes within the bacterial genome, a process that can be divided into three steps (Fig 1).

*1*. ***Similarity search***. The coding sequence of each gene is extracted from the bacterial genomic sequence according to the coordinates in the user provided GFF file, and then translated into its corresponding protein sequence. Each protein sequence is then searched against the ProphET reference database to identify phage-like matches (e-value < 10^−5^).

**Fig 2:**
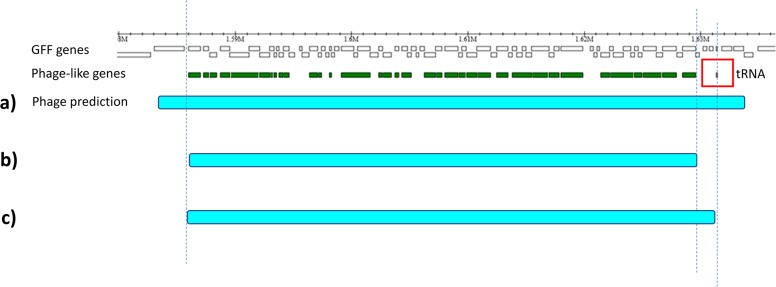
Refining prophage edges. The ruler on top indicates bacterial genome coordinates. White boxes correspond to annotated genes present in the GFF file provided by the user, while green boxes correspond to phage-like genes assigned by ProphET. Cyan horizontal bars depict iterative adjustments by ProphET to the boundaries of the predicted prophage. After preliminary prophage prediction **(a)**, borders are trimmed to the first and last phage-like gene within those boundaries **(b)**. Next, ProphET searches for tRNA genes within a 3 kb region upstream and downstream of the predicted prophage ends. If tRNA genes are found, phage borders are extended or contracted to match the outwards coordinates of the tRNA genes **(c)**.

2. ***Prophage density calculation*.** The number of phage-like genes is calculated within a sliding window of length 10 kb, moving in increments of 1 kb across the genome sequence. Instances with at least eight phage-like genes and an overall length longer than half of the sliding window length (5 kb) are selected. Overlapping windows are merged and assigned as preliminary prophage predictions. The length of the sliding window and the minimum number of phage-like genes in each window were defined according to the distribution of these traits for the bacteriophage genomes in the ProphET database: only 6.2% of phage genomes are shorter than 10 kb, and even those have more than 8 genes (S1 Fig).

3. ***Edge refinement***. Finally, ProphET trims the borders of preliminary prophage predictions at the last phage-like gene. As tRNA genes are hotspots of prophage integration [19], ProphET searches 3 kb upstream and downstream of the putative prophage borders for tRNA genes. If a tRNA gene is found, it extends or contracts the prophage predicted border accordingly, resulting in the final prophage prediction (Fig 2).

### ProphET output files

ProphET outputs i) a file listing the bacterial genome coordinates of each predicted prophage in BED format, ii) an image depicting the predicted prophage positions in the bacterial genome, and iii) FASTA files with the nucleotide sequence of each predicted prophage. ProphET also outputs a folder with intermediate files used in prophage predictions, as the BLAST results.

### ProphET performance evaluation

The performance of ProphET was evaluated and compared to four other published prophage prediction tools, Prophinder, PHAST, PHASTER and PhySpy, using 54 well-studied bacterial genomes containing 267 prophages, manually annotated by Casjens [5], serving as a gold standard (S1 Table). This was the same dataset previously used as the gold standard in publications reporting on Prophinder v0.4 and PHAST. All of these programs were run with default parameters, with exception of PhySpy which was run with both a generic (default) training set as well as a taxonomically-informed choice of training set. PhySpy version 3.2 currently has 31 training sets available, comprising 30 bacterial species from 21 different families and one “generic” default training set (S2 Table). If available, a training set from the same species as the evaluated genome was selected (this was the case for 38 of the 54 genomes in our gold standard set). Otherwise, a training set from the same family was selected (this was the case for 7 of the 54 genomes). For the 9 remaining genomes in our gold standard set, neither of these conditions was met, and the test set was evaluated using the generic test set (S3 Table).

**Fig 3:**
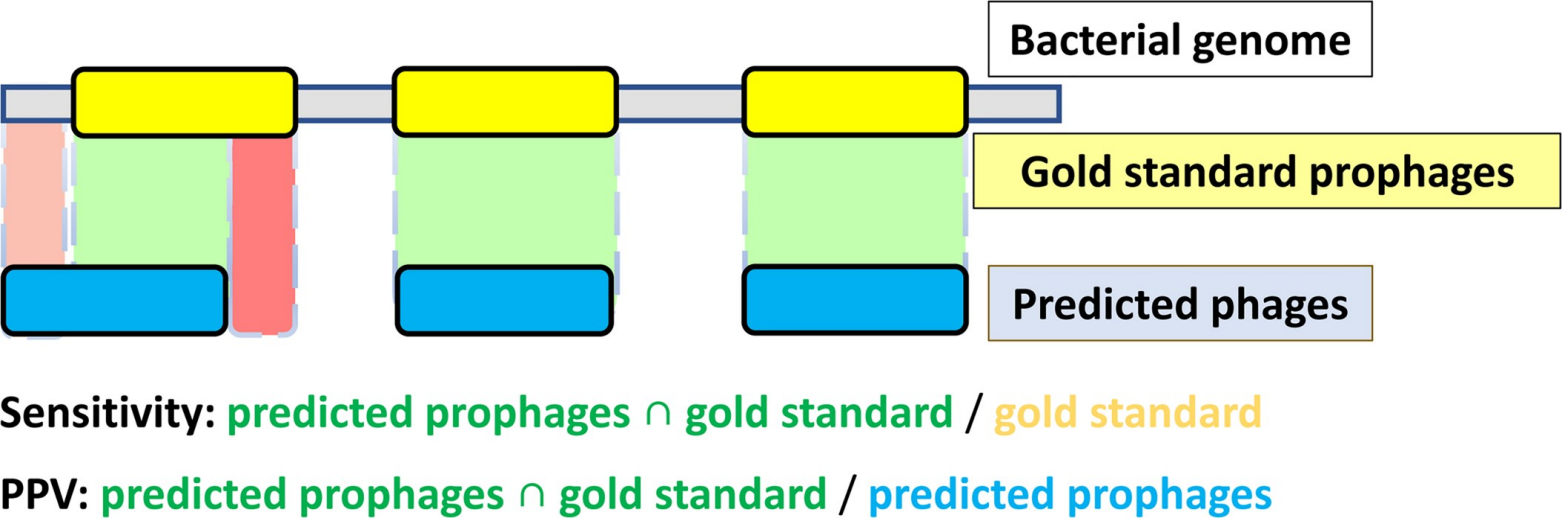
Prophage prediction performance evaluation methodology. Grey bar corresponds to the bacterial genome evaluated, while the yellow and blue boxes correspond to gold standard prophage annotations and the predicted prophages, respectively. The gold standard consists of 267 prophages in 54 bacterial genomes manually annotated by Casjens 2003. Sensitivity was calculated by dividing the length of the intersection between the gold standard and predicted prophages (green regions) by the length of the gold standard prophages (yellow regions). PPV was calculated by dividing the same intersection (green regions) by the length of the predicted prophages (blue regions). Light and dark red areas indicate false positives and false negative regions, respectively.

In order to evaluate the results of our benchmarking, we considered as true positives (TP) the overall length of the overlap between prophage predictions and the gold standard; false positives (FP) as the overall length of predictions that did not overlap with the gold standard; and false negatives (FN) as the overall length of regions of the gold standard that were not included in the predictions (Fig 3). The sensitivity (TP/TP+FN) and positive predictive value (PPV = TP/TP+FP) were computed and compared to those obtained by Prophinder, PHAST, PHASTER and PhySpy. To compare scalability, we benchmarked run times for the five prophage prediction tools using the same set of 54 genomes, measuring the time elapsed between the submission of the first genome and the receipt of results for all prophage predictions.

## Results and discussion

In order to efficiently predict phages from large datasets of bacterial genomes, we designed ProphET, a fast, easy to use, and highly scalable tool that can accurately identify bacteriophages in complete and draft genome sequences. ProphET searches the predicted proteome from an input genome sequence against a database of phage genes automatically downloaded from NCBI-GenBank (see Materials and Methods). ProphET requires that the user provide pre-computed gene annotations as input, permitting the user to use their preferred annotation tool as well as to remove known genes that could lead to incorrect predictions of prophages, if applicable. Since prophage predictions are based on the density of phage genes, fragmented assemblies and partial gene annotation files could result in missed prophage predictions. We recommend the use of complete genomes and GFF annotation files rather than draft assemblies and partial gene annotation files; however, ProphET can identify prophages wholly contained within a contig from a fragmented assembly.

We compared the performance of ProphET to four phage prediction tools, Prophinder v0.4 [13], PHAST [14], PHASTER [15], and PhySpy [16] (see Materials and Methods). ProphET had the highest overall performance among the four programs (Table 1). The slightly higher sensitivity obtained by PHAST, PHASTER and PhySpy (77.7% for PHAST, 78.1% for PHASTER and 79% for PhySpy, versus 73.3% for ProphET) came at the cost of a loss in PPV (69.2% for PHAST, 74.6% for PHASTER and 55.8% for PhySpy, versus 84.2% for ProphET). The higher PPV obtained by ProphET is a consequence of its lower false positive rate when compared to the other predictors. The majority of the prophages missed by ProphET (false negatives) were also not detected by the other predictors. These mainly consisted of short prophages without phage-like genes, according to our reference database. This limitation may be addressed as more phages are annotated and included in reference databases.

**Table 1:** Prophinder, PHAST, PHASTER, ProphET and PhySpy sensitivity and PPV.

	Sensitivity	PPV
**Prophinder**	69.8	73.5
**PHAST**	77.0	69.2
**PHASTER**	78.1	74.6
**ProphET**	73.3	84.2
**PhySpy**	79.0	47.8
PhySpy*	77.7	55.8

High and low values are represented on a scale from red to blue. PhySpy: Predictions using the generic training set. PhySpy*: Predictions using a taxonomically-optimized training set (Materials and Methods; S3 Table).

**Fig 4:**
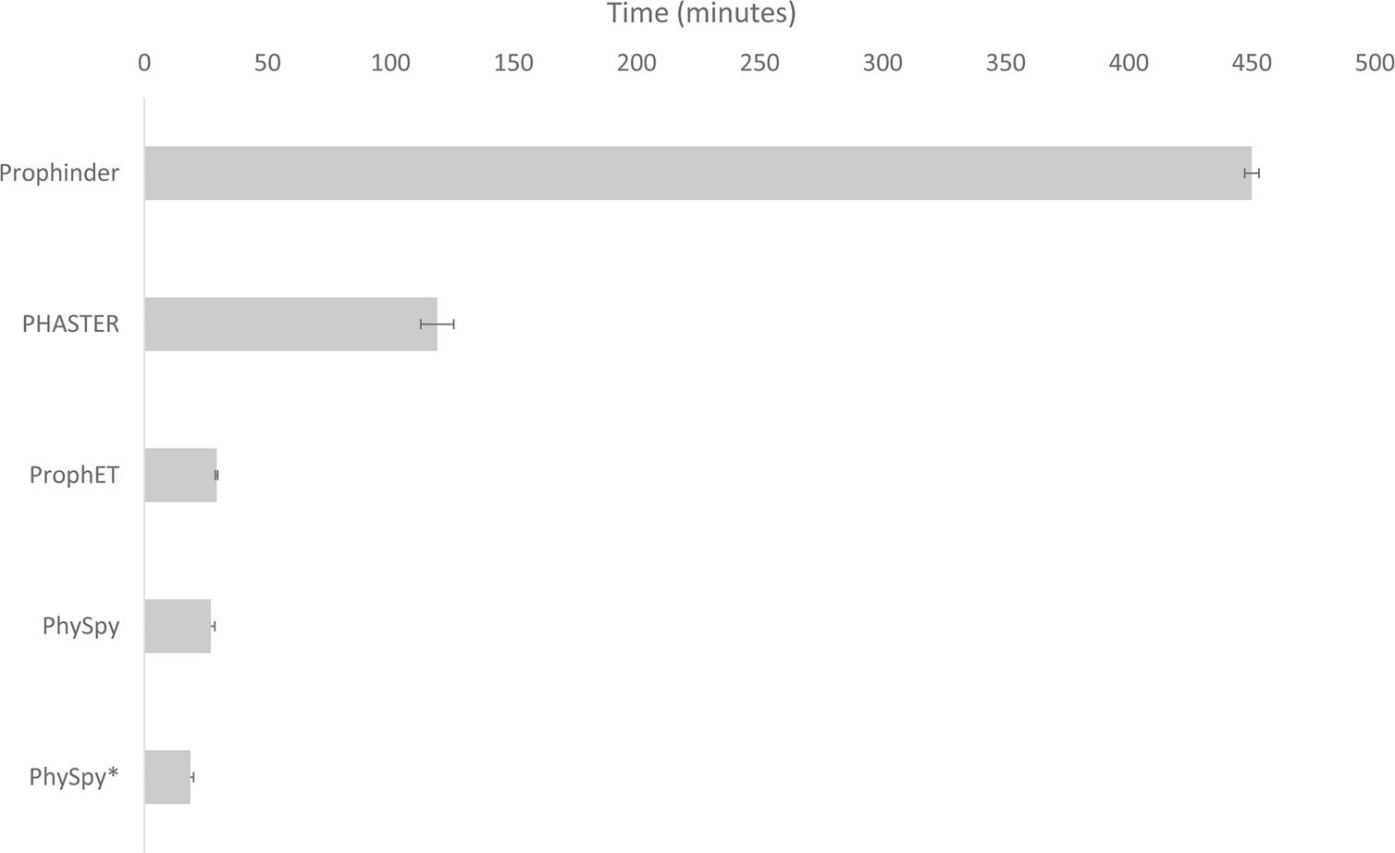
ProphET runs faster than web-based tools and with comparable speed to PhySpy. The run time required is shown (in minutes) for Prophinder, PHASTER, ProphET, PhySpy (generic training set) and PhySpy* (taxonomically optimized training set) to perform prophage identification for our test set of 54 well-studied bacterial genomes (S1 Table). Three separate evaluations were performed on different days.

Finally, to compare scalability, we benchmarked run times for Prophinder, PHASTER, PhySpy and ProphET using the same set of 54 genomes. As PHAST is an older version of PHASTER with lower sensitivity and specificity, it was not evaluated. We measured the time elapsed between the submission of the first genome and the receipt of results for all prophage predictions. In our comparison, ProphET was four times faster than PHASTER, over 15 times faster than Prophinder, and comparable in speed to PhySpy (Fig 4, S4 Table). The benchmarking of the standalone applications ProphET and PhySpy was performed using 50 processors; however, their run times could be further reduced if more powerful servers or cloud computing were used. Using a single processor to analyze the 2 MB genomic sequence of *Lactobacillus johnsonii* NCC 533 (NC_005362.1), ProphET took 12 minutes, with a peak memory usage of 70 MB of RAM on a desktop computer with 1.4 GHz Intel Core i5 processor and 8 GB 1600 MHz DDR3 RAM. In addition, in our benchmarking ProphET ran in an automated fashion without user intervention, whereas we had to manually re-submit around 10% of the genomes to PHASTER due to dropped server connections.

## Conclusion

ProphET is a fast and scalable, easy to install and to use program to identify prophage sequences in bacterial genomes. In our benchmarking, ProphET ran faster or with comparable speed with current used prophage predictors, with comparable sensitivity and substantially better precision. Unlike the current most widely-used prophage predictors which are web-based, ProphET runs as a stand-alone application and scales well with large datasets, a very desirable characteristic as projects requiring the sequence and annotation of thousands of bacterial genomes are becoming routine. Therefore, we believe that ProphET will be a great asset for the large-scale identification of prophage within rapidly expanding genomic datasets.

## Supporting information

S1 Fig Distribution of phage sizes and the number of genes per phage in the ProphET reference database. **a)** Distribution of phage genome size, **b)** number of genes per phage and **c)** the most relevant percentiles among the 1,435 phages in ProphET database. The 95% percentile indicates that 95% of the evaluated phages had less than 270 genes.
(DOCX)

S1 Table Bacterial genomes used as a gold standard in the evaluation of performance. This table contains links to the 54 bacterial genomic sequences used as gold standard in the evaluation of performance of Prophinder, PHAST, PHASTER, and ProphET.
(XLSX)

S2 Table Training sets available to PhySpy. PhySpy provides a total of 31 training sets, comprising 30 bacterial species from 21 different families and one “generic” default training set.
(XLSX)

S3 Table Training sets used in each PhySpy run. PhySpy prophage prediction was performed using a training set from the same species or family as the test genome, when available. Otherwise, the prediction was performed with the generic test set.
(XLSX)

S4 Table Run-time evaluation of prophage prediction programs. The total elapsed time to perform prophage predictions in the 54 bacterial genomes was measured on three different days, represented by the “Round” columns. PhySpy: Predictions using the generic training set. PhySpy*: Predictions using a taxonomically optimized training set (Materials and Methods; S3 Table).
(XLSX)

## Abbreviations

FP: False positives
FN: False negatives
GFF: General Feature Format
NIAID: National Institute of Allergy and Infectious Diseases
NCBI: National Center for Biotechnology Information
PHAST: Phage Search Tool
PHASTER: Phage Search Tool Enhanced Release
PPV: Positive Predictive Value
ProphET: Prophage Estimation Tool
TP: True Positives

## References

[1] Brüssow H, Canchaya C, Hardt W, Bru H. Phages and the Evolution of Bacterial Pathogens: from Genomic Rearrangements to Lysogenic Conversion Phages and the Evolution of Bacterial Pathogens: from Genomic Rearrangements to Lysogenic Conversion. Microbiol Mol Biol Rev. 2004;68(3):560–602. 10.1128/MMBR.68.3.560-602.2004 15353570 PMC515249

[2] Davies E V., Winstanley C, Fothergill JL, James CE. The role of temperate bacteriophages in bacterial infection. FEMS Microbiol Lett. 2016;363(5):1–10. 10.1093/femsle/fnw015 26825679

[3] Faruque SM, Mekalanos JJ. Phage-bacterial interactions in the evolution of toxigenic *Vibrio cholerae*. Virulence. 2012;3(7):556–65. Available from: http://www.tandfonline.com/doi/abs/10.4161/viru.22351 23076327 10.4161/viru.22351 PMC3545932

[4] Aziz RK, Edwards RA, Taylor WW, Low DE, McGeer A, Kotb M. Mosaic prophages with horizontally acquired genes account for the emergence and diversification of the globally disseminated M1t1 clone of *Streptococcus pyogenes*. J Bacteriol. 2005;187(10):3311–8. 10.1128/JB.187.10.3311-3318.2005 15866915 PMC1112019

[5] Casjens S. Prophages and bacterial genomics: What have we learned so far? Vol. 49, Molecular Microbiology. 2003 p. 277–300. 10.1046/j.1365-2958.2003.03580.x 12886937

[6] Laing CR, Zhang Y, Gilmour MW, Allen V, Johnson R, Thomas JE, et al A comparison of Shiga-Toxin 2 bacteriophage from classical enterohemorrhagic *Escherichia coli* serotypes and the German E. coli O104:H4 outbreak strain. PLoS One. 2012;7(5). 10.1371/journal.pone.0037362 PMC3359367 22649523

[7] Yan X, Fratamico PM, Bono JL, Baranzoni GM, Chen CY. Genome sequencing and comparative genomics provides insights on the evolutionary dynamics and pathogenic potential of different H-serotypes of Shiga toxin-producing *Escherichia coli* O104. BMC Microbiol. 2015;15(1). 10.1186/s12866-015-0413-9 PMC4393859 25887577

[8] Grose JH, Casjens SR. Understanding the enormous diversity of bacteriophages: The tailed phages that infect the bacterial family Enterobacteriaceae. Virology. 2014;468:421–43. 10.1016/j.virol.2014.08.024 25240328 PMC4301999

[9] Levin BR, Bull JJ. Population and evolutionary dynamics of phage therapy. Nat Rev Microbiol. 2004;2(2):166–73. 10.1038/nrmicro822 15040264

[10] Hendrix RW. Bacteriophages: Evolution of the Majority. Vol. 61, Theoretical Population Biology. 2002 p. 471–80. 12167366 10.1006/tpbi.2002.1590

[11] Suttle C a. Marine viruses—major players in the global ecosystem. Nat Rev Microbiol. 2007;5(10):801–12. 10.1038/nrmicro1750 17853907

[12] Pope WH, Bowman CA, Russell DA, Jacobs-Sera D, Asai DJ, Cresawn SG, et al Whole genome comparison of a large collection of mycobacteriophages reveals a continuum of phage genetic diversity. Elife. 2015 1;4:e06416 10.7554/eLife.06416 25919952 PMC4408529

[13] Lima-Mendez G, Van Helden J, Toussaint A, Leplae R. Prophinder: A computational tool for prophage prediction in prokaryotic genomes. Bioinformatics. 2008;24(6):863–5. 10.1093/bioinformatics/btn043 18238785

[14] Zhou Y, Liang Y, Lynch KH, Dennis JJ, Wishart DS. PHAST: A Fast Phage Search Tool. Nucleic Acids Res. 2011;39(SUPPL. 2). 10.1093/nar/gkr485 PMC3125810 21672955

[15] Arndt D, Grant JR, Marcu A, Sajed T, Pon A, Liang Y, et al PHASTER: a better, faster version of the PHAST phage search tool. Nucleic Acids Res. 2016;44(W1):W16–21. 10.1093/nar/gkw387 27141966 PMC4987931

[16] Akhter S, Aziz RK, Edwards RA. PhiSpy: A novel algorithm for finding prophages in bacterial genomes that combines similarity-and composition-based strategies. Nucleic Acids Res. 2012;40(16). 10.1093/nar/gks406 PMC3439882 22584627

[17] Krupovic M, Prangishvili D, Hendrix RW, Bamford DH. Genomics of bacterial and archaeal viruses: dynamics within the prokaryotic virosphere. Microbiol Mol Biol Rev. 2011 12;75(4):610–35. 10.1128/MMBR.00011-11 22126996 PMC3232739

[18] Davidson AL, Dassa E, Orelle C, Chen J. Structure, function, and evolution of bacterial ATP-binding cassette systems. Microbiol Mol Biol Rev. 2008;72(2):317–64, table of contents. Available from: http://www.pubmedcentral.nih.gov/articlerender.fcgi?artid=PMC2415747 10.1128/MMBR.00031-07 18535149 PMC2415747

[19] Campbell A. Prophage insertion sites. Vol. 154, Research in Microbiology. 2003 p. 277–82. 10.1016/S0923-2508(03)00071-8 12798232

