# Supplementary material for: ProphET, prophage estimation tool: A stand-alone prophage sequence prediction tool with self-updating reference database
Source: PLoS One. 2019 Oct 2;14(10):e0223364. doi: 10.1371/journal.pone.0223364 (PMC6774505; doi:10.1371/journal.pone.0223364)
Supplement: S1 Fig — a) Distribution of phage genome size, b) number of genes per phage and c) the most relevant percentiles among the 1,435 phages in ProphET database. The 95% percentile indicates that 95% of the evaluated phages had less than 270 genes. (DOCX) [file pone.0223364.s001.docx]

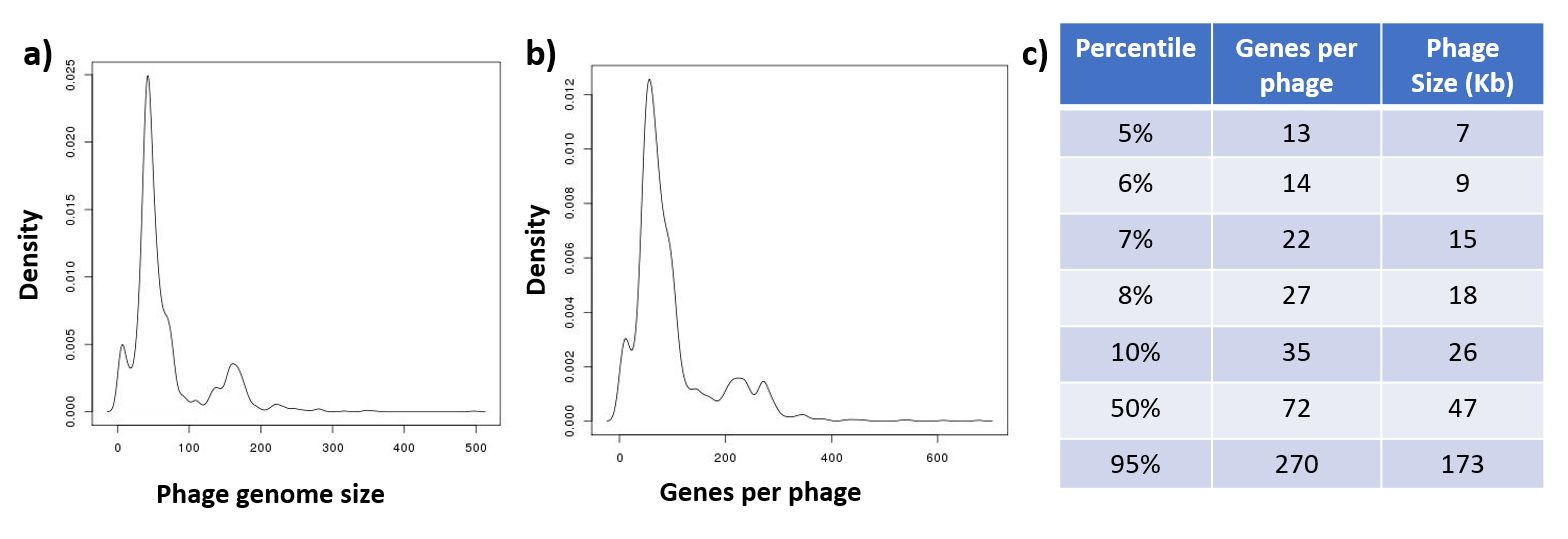


**S1 Figure. Distribution of phage sizes and the number of genes per phage in the ProphET reference database**. **a)** Distribution of phage genome size, **b)** number of genes per phage and **c)** the most relevant percentiles among the 1,435 phages in ProphET database. The 95% percentile indicates that 95% of the evaluated phages had less than 270 genes.
